# Alcohol Intake and Hypertensive Disorders of Pregnancy: A Negative Control Analysis in the ALSPAC Cohort

**DOI:** 10.1161/JAHA.121.025102

**Published:** 2022-09-29

**Authors:** Florence Z. Martin, Abigail Fraser, Luisa Zuccolo

**Affiliations:** ^1^ MRC Integrative Epidemiology Unit (IEU) University of Bristol United Kingdom; ^2^ Department of Population Health Sciences, Bristol Medical School University of Bristol United Kingdom; ^3^ NIHR Biomedical Research Centre, Bristol Medical School University of Bristol United Kingdom

**Keywords:** alcohol, ALSPAC, gestational hypertension, negative control, preeclampsia, pregnancy

## Abstract

**Background:**

Alcohol intake increases blood pressure yet estimates of associations between maternal intake and hypertensive disorders of pregnancy (HDP) are sparse and range from null to a protective effect. Here we estimated the association of maternal drinking during pregnancy with preeclampsia and gestational hypertension (separately and jointly, as HDP). We used partner's alcohol intake as a negative control exposure, beverage type‐specific models, and a range of sensitivity analyses to strengthen causal inference and reduce the influence of bias.

**Methods and Results:**

We performed a longitudinal analysis of prospectively collected data on self‐reported alcohol intake and presence of HDP from the UK ALSPAC (Avon Longitudinal Study of Parents and Children) cohort. Multivariable multinomial regression models were adjusted for confounders and mutually adjusted for partner's or maternal alcohol intake in the negative control analysis. We also performed a beverage type analysis of the effect of beer and wine separately on HDP risk, owing to different social patterning associated with different drinks. Sensitivity analyses assessed the robustness of results to assumptions of no recall bias, no residual confounding, and no selection bias. Of the 8999 women eligible for inclusion, 1490 fulfilled the criteria for HDP (17%). Both maternal and partner's drinking were associated with decreased HDP odds (mutually adjusted odds ratio [OR], 0.86; [95% CI, 0.77–0.96], *P*=0.008 and OR, 0.82; [95% CI, 0.70–0.97], *P*=0.018, respectively). We demonstrate the validity of the negative control analyses using the same approach for smoking as the exposure. This confirmed an inverse association for maternal but not partner's smoking, as expected. Estimates were more extreme for increasing levels of wine intake compared with increasing levels of beer. Multiple sensitivity analyses did not alter our conclusions.

**Conclusions:**

We observed an inverse relationship between alcohol intake during pregnancy and risk of HDP for both maternal and, more surprisingly, partner's drinking. We speculate that this is more likely to be due to common environmental exposures shared between pregnant women and their partners rather than a true causal effect. This warrants further investigation using different study designs, including Mendelian randomization.

**Nonstandard Abbreviations and Acronyms**
ALSPAC: Avon Longitudinal Study of Parents and Children
HDP: hypertensive disorders of pregnancy
SEP: socioeconomic position

**CLINICAL PERSPECTIVE**

**What Is New?**

- Maternal alcohol intake during pregnancy was associated with a decreased risk of developing hypertensive disorders of pregnancy, namely gestational hypertension and preeclampsia, and this association was robust to a range of sensitivity analyses.
- Partner's alcohol intake during pregnancy was also associated with a decreased risk of maternal hypertensive disorders of pregnancy, including after mutual adjustment for maternal alcohol intake.

**What Are the Clinical Implications?**

- Findings suggest that the inverse association between alcohol intake and hypertensive disorders of pregnancy is unlikely to reflect a causal effect and is more likely to be driven by unmeasured confounding shared between women and their partners.
- Given the evidence that alcohol is fetotoxic and overall detrimental to cardiovascular health, advice about alcohol use during pregnancy should continue to recommend abstention to minimize any immediate or long‐term harm.

Hypertensive disorders of pregnancy (HDP) is an umbrella term for gestational hypertension and preeclampsia, both characterized by de novo hypertension arising during pregnancy, with concurrent proteinuria in preeclampsia.^1^ There are several known risk factors for the development of HDP, screened for at the antenatal booking appointment, including older maternal age, obesity, history of HDP, and diabetes.^2^ Although alcohol intake is known to increase blood pressure,^3^, ^4^, ^5^, ^6^ previous studies have produced inconsistent results regarding the risk of HDP when comparing women consuming alcohol in pregnancy to those abstaining.^7^, ^8^, ^9^, ^10^

In the absence of randomized controlled trials or natural experiments investigating the role of alcohol on HDP, relevant evidence comes entirely from observational studies. Residual confounding by factors such as socioeconomic position and smoking is a concern, because smoking and drinking alcohol are correlated^11^ and socially patterned, and smoking during pregnancy is associated with a lower risk of developing preeclampsia.^12^ Therefore, failure to adequately account for smoking in analyses of the association between prenatal alcohol and preeclampsia and HDP could lead to biased estimates in the same direction as the smoking‐HDP effect.

A recent (not currently peer‐reviewed) systematic review showed some evidence of an inverse association between alcohol use in pregnancy and preeclampsia, especially when examining prospective studies (pooled odds ratio [OR], 0.64; [95% CI, 0.54–0.76]).^13^ The evidence pointing to an inverse association is paradoxical given the blood pressure‐elevating effect of alcohol intake outside of pregnancy.

Negative control designs can be used in observational epidemiological studies to elucidate whether an association is likely to be causal or whether it is a result of unmeasured or residual confounding.^14^ For studies examining exposures during pregnancy, partner behaviors can be used as the negative control exposure for maternal outcomes. This is based on the assumption that partner's alcohol intake should not cause maternal HDP. If an association is observed, it suggests a common confounding structure by shared environment.

In this study, we aimed to quantify the association between alcohol intake during pregnancy and HDP in a large population‐based prospective cohort—ALSPAC (Avon Longitudinal Study of Parents and Children). We employed a negative control exposure design, using partners' alcohol intake during pregnancy, to detect the presence of confounding and disentangle association from causation. We also performed a beverage type analysis of the effect of beer and wine separately on HDP risk, owing to different social patterning associated with different drinks, and a range of sensitivity analyses to increase confidence in our findings.

## METHODS

Because of the sensitive nature of the data collected for this study, requests to access the data set from qualified researchers may be sent to the ALSPAC Executive Committee at https://proposals.epi.bristol.ac.uk/. Source code available from https://github.com/flozoemartin/MP2.

### Study Population

**Figure 1:**
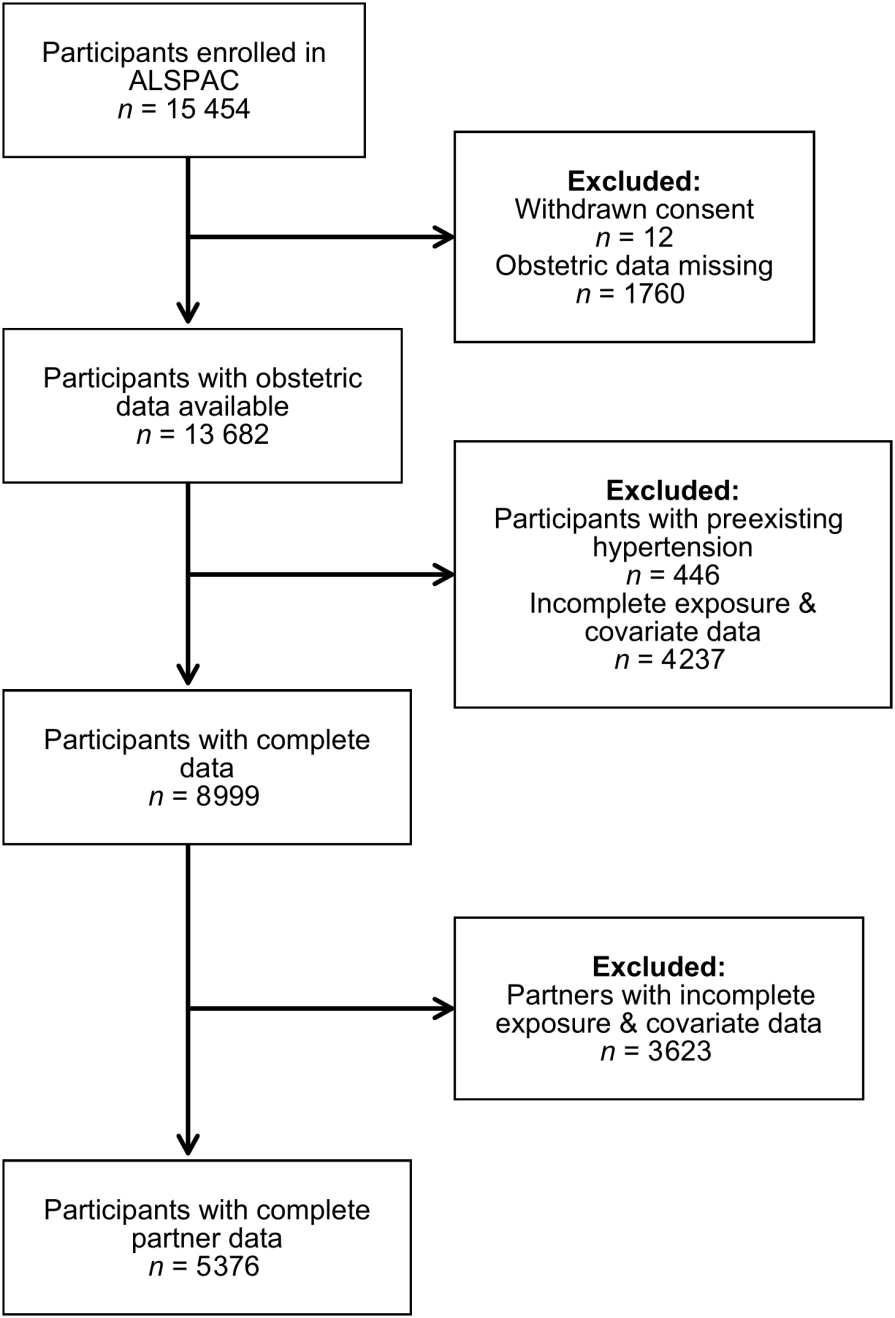
Flow of participants through the study. ALSPAC indicates Avon Longitudinal Study of Parents and Children.

We used information from the ALSPAC cohort to define the study population in this analysis. ALSPAC is a UK‐based cohort of 15 454 women recruited in the early 1990s from the Southwest of England and followed up pre‐ and postnatally via self‐report questionnaires and in‐person clinics.^15^ Previous publications have described the maternal cohort in full.^16^ Please note that the study website contains details of all the data that are available through a fully searchable data dictionary and variable search tool (http://www.bristol.ac.uk/alspac/researchers/our‐data/).^17^ For transparency, we did not preregister this study on Open Science Framework. We included mothers with self‐report questionnaire data on alcohol intake during pregnancy and other covariates deemed to be potential confounders, as well as obstetric data abstracted from medical records (n=8999) (Figure 1).

Ethical approval for this study was secured from the ALSPAC Ethics and Law Committee and local Research Ethics Committee (North Somerset and South Bristol). Participants gave consent for their obstetric data to be abstracted and answers to self‐report questionnaires to be used in subsequent research; individuals have the right to withdraw from the ALSPAC cohort at any time during follow‐up.

### Measures

#### Alcohol Intake During Pregnancy

The exposure in this study, alcohol intake during pregnancy, was measured using multiple questionnaires sent prenatally and in the immediate postpartum period. At around 18 weeks' gestation, participants were asked how often they drank alcohol: (1) in the first 3 months of pregnancy and (2) since the baby first moved. These questions were categorized as none, <1 drink per week, 1+ glasses per week, 1 to 2 glasses per day, 3 to 9 glasses per day, and 10+ glasses per day. They were also asked about how much of each type of drink (beer, wine, spirits, or other) they drank on a typical day, having been advised that a glass was the equivalent of a half pint (beer), a wineglass (wine), or a pub measure (spirit). The questionnaire that was sent at the same time to partners asked the same questions regarding alcohol intake. After birth, mothers and partners were asked about average alcohol intake in the final 2 months of pregnancy, using the same categories. Using the answers given in both questionnaires, the maximum amount of alcohol that each participant reported to drink at any time during pregnancy was used to categorize women: none, low to moderate (1–6 drinks per week), and heavy (≥7 drinks per week). For example, a participant reporting heavy drinking since the baby first moved (questionnaire B) and no drinking in the last 2 months of pregnancy (questionnaire E) would have been categorized as a heavy drinker in this analysis.

At 18 weeks' gestation, both mothers and partners were asked how many days in the past month they had consumed the equivalent of 2 pints of beer or more. Although this does not perfectly align with other definitions of binge drinking^18^ including the National Institute for Alcohol Abuse and Alcoholism's definition (≥4 drinks in 2 hours),^19^ it provided an appropriate additional category for sensitivity analyses to separate those in the “heavy” drinking category who were not bingeing from those who were drinking multiple alcoholic beverages in 1 day.

Given the specific questions asked at 18 weeks' gestation pertaining to the intake of different types of alcoholic beverages at the time of the questionnaire being filled out, we derived 2 variables for beer and wine intake during pregnancy. In other words, the beer drinker group consisted of those who had not reported wine consumption and vice versa for wine. We used the same categorization of amounts drunk as the primary analysis (none, low to moderate, and heavy) for each beverage type. Reporting of spirits/other alcohol intake and bingeing was then compared in beer and wine groups to better understand overall drinking patterns in these 2 groups.

#### Hypertensive Disorders of Pregnancy

For women who gave informed consent to have their obstetric data abstracted, all recorded measurements of both systolic and diastolic blood pressure were obtained, as well as events of proteinuria, as previously described in detail.^20^ Briefly, all measurements were collated by research midwives and the 1988 International Society for the Study of Hypertension in Pregnancy criteria^1^ definitions were superimposed onto measurements for each participants. Thus, women were categorized as normotensive, gestational hypertension, or preeclampsia. As shown in Figure 1, women with existing hypertension were excluded (n=446/12 010), as the definitions of HDP used specify “incidence of hypertension during pregnancy.”

#### Other Variables

Covariates for this analysis were defined a priori using evidence from the literature to support a potential relationship with both the exposure and the outcome: maternal age at delivery, maternal race or ethnicity, maternal body mass index (BMI), smoking status (before and during pregnancy), maternal socioeconomic position (SEP), marital status, and parity. Women reported their age, race or ethnicity, height and weight (used to calculate prepregnancy BMI), smoking habits, educational attainment (proxy for SEP), marital status, and parity on self‐completed questionnaires sent out during pregnancy.

Three questionnaires asked participants about their smoking habits at different times during pregnancy: at 18 weeks' gestation women were asked about smoking early in pregnancy and current smoking, at 32 weeks' gestation current smoking habits were described, and at 8 week's postpartum participants reported their smoking habits in the last 2 months of pregnancy. Two smoking variables were generated: a binary variable for any or no smoking during pregnancy and a categorical variable for average number of cigarettes smoked per day during pregnancy.

All the variables described (except prepregnancy smoking) were also measured via self‐report questionnaire for the partners of participants, which were abstracted for adjustment of the negative control analysis. Partner's smoking status was measured across several variables in 2 prenatal questionnaires, which were collated to create a binary variable of any or no smoking during their partner's pregnancy.

HDP is associated with other pregnancy complications including diabetes,^21^, ^22^ kidney disease,^23^ rheumatoid arthritis,^24^ and multiple pregnancy.^22^ Diabetes noted during pregnancy (both preexisting and gestational) and multiple pregnancies were abstracted from obstetric records; kidney disease, both recent and historic diagnoses, was self‐reported at 12 weeks' gestation. Rheumatoid arthritis during pregnancy was not available in ALSPAC; however, any arthritis, both recent and historic, was self‐reported during pregnancy (12 weeks' gestation).

### Statistical Analysis

Women's characteristics were described by levels of alcohol intake in pregnancy using means (SDs) for continuous variables and percentages for binary variables. There was no evidence of an association between HDP (outcome) and study attrition, and all covariates had <15% missing data. Thus, we deemed that multiple imputation would not increase the study efficiency in this case and the use of a complete case analysis was the most appropriate approach^25^, ^26^ (Tables S1 through S4).

For the primary analysis, we used multivariable logistic regression to estimate the OR of HDP by increasing categories of alcohol intake (none, low to moderate, and heavy drinking). Because of the 3‐level exposure variable, likelihood‐ratio tests were used to test for dose–response, comparing alcohol use as a single 3‐level (continuous) variable (model A) or including alcohol as 2 dummy variables (model B). We used multivariable multinomial logistic regression models to estimate the relative risk ratio of developing gestational hypertension and preeclampsia compared with normotensive, using the outcome over 3 categories. Both of these models were also mutually adjusted for their partner's alcohol intake for comparison with the negative control analysis.

The primary analysis was then repeated using partners' alcohol intake as the exposure. The comparison of maternal and partner's association with HDP rested on the assumption that mothers and partners share environmental and behavioral factors affecting or correlating with their alcohol drinking that also affect maternal HDP risk, but only maternal alcohol use could physiologically affect HDP risk. Both adjusted and mutually adjusted models were fitted, with the latter additionally adjusting for mother's alcohol intake to account for the potential bias from assortative mating.^27^ We additionally report the association of maternal and partner's smoking during pregnancy with risk of HDP, with similar mutual adjustments. Smoking during pregnancy was used as a supplementary exposure in the negative control model to check our prior assumption that a maternal exposure with evidence of an association with HDP, such as maternal smoking, should indeed be associated with HDP but that partner exposure would not.

To further evaluate the role of residual confounding by SEP or associated factors, we compared estimates of the association of HDP risk with wine and beer drinking separately. This was done under the assumption that intake of these 2 beverages follow different SEP patterning, as previously demonstrated in this cohort.^28^ It follows therefore that consistent results would strengthen a causal interpretation, whereas discordant results could point to confounding biasing the findings.

We conducted sensitivity analyses to assess to what extent estimates obtained from the primary analysis were robust to sources of bias including (1) excluding women who experienced pregnancy complications associated with HDP (diabetes, kidney disease, arthritis, or multiple pregnancy), (2) using a categorical smoking covariate in the model (as opposed to binary) to better account for residual confounding by smoking, (3) excluding those women who responded to alcohol‐related questions after 20 weeks' gestation to limit recall bias (HDP status influencing reporting of the exposure), and (4) excluding women who abstained from alcohol before pregnancy to limit the potential impact of existing ill health.

## RESULTS

### Study Sample

After exclusions, 8999 women (58% of the whole sample) were eligible for inclusion in this study (Figure 1), of whom 1490 fulfilled the criteria for HDP (17% of the eligible sample). Table shows the characteristics of included participants, by amounts of alcohol intake during pregnancy. Those who reported low‐to‐moderate drinking were older, more highly educated, more likely to be White, and had a lower BMI compared with those who reported no alcohol intake during pregnancy. Compared with non‐drinkers, heavy drinkers were also more likely to be older, White, and more highly educated; heavy drinkers were also more likely to smoke both before and during pregnancy, had more children, and were less likely to be married (Table). When comparing characteristics of participants who developed HDP with those who remained normotensive during pregnancy, those with HDP had a higher mean BMI and were less likely to be multiparous (Table S5).

**Table 1:** Maternal Characteristics in the Complete Case Cohort (n=8999) and Partner Characteristics in the Negative Control Cohort (n=5376) by Categories of Alcohol Intake During Pregnancy

Characteristic	Alcohol intake	Maternal data (complete case cohort)	No. (%) (unless otherwise specified)	Partner data (negative control cohort)	No. (%) (unless otherwise specified)
Age at delivery, mean, y (SD)	None	2415	27.7 (4.7)	141	30.0 (6.2)
	Low to moderate	4696	28.9 (4.4)	3943	30.5 (5.4)
	Heavy	1888	28.7 (4.9)	1292	32.0 (5.6)
Body mass index prepregnancy, mean, kg/m^2^ (SD)	None	2415	23.0 (4.0)	141	24.6 (5.0)
	Low to moderate	4696	22.7 (3.6)	3943	25.0 (3.8)
	Heavy	1888	23.1 (3.6)	1292	24.8 (3.8)
Any smoking prepregnancy	None	2415	705 (29.2)	…	…
	Low to moderate	4696	1299 (27.7)	…	…
	Heavy	1888	874 (46.3)	…	…
Any smoking during pregnancy	None	2415	552 (22.9)	141	45 (31.9)
	Low to moderate	4696	991 (21.1)	3943	1257 (31.9)
	Heavy	1888	753 (39.9)	1292	513 (39.7)
Multiparous (18 wk gestation)	None	2415	1280 (53.0)	141	75 (55.3)
	Low to moderate	4696	2589 (55.1)	3943	2088 (53.0)
	Heavy	1888	1124 (59.5)	1292	630 (48.8)
Black, Asian, and other non‐Caucasian ethnicities (32 wk gestation)	None	2415	61 (2.5)	141	7 (5.0)
	Low to moderate	4696	80 (1.7)	3943	61 (1.6)
	Heavy	1888	24 (1.3)	1292	17 (1.3)
University degree (32 wk gestation)	None	2415	232 (9.6)	141	30 (21.3)
	Low to moderate	4696	792 (16.9)	3943	792 (20.1)
	Heavy	1888	221 (11.7)	1292	409 (31.7)
Married (8 wk gestation)	None	2415	1904 (78.8)	141	118 (83.7)
	Low to moderate	4696	3792 (80.8)	3943	3317 (84.1)
	Heavy	1888	1303 (69.0)	1292	1070 (82.8)

Among partners, heavy drinkers during pregnancy were more likely to be older and White compared with nondrinkers; heavy drinkers were also more likely to smoke during pregnancy and more likely to have a degree than nondrinkers.

### Maternal Alcohol Intake and HDP

**Figure 2:**
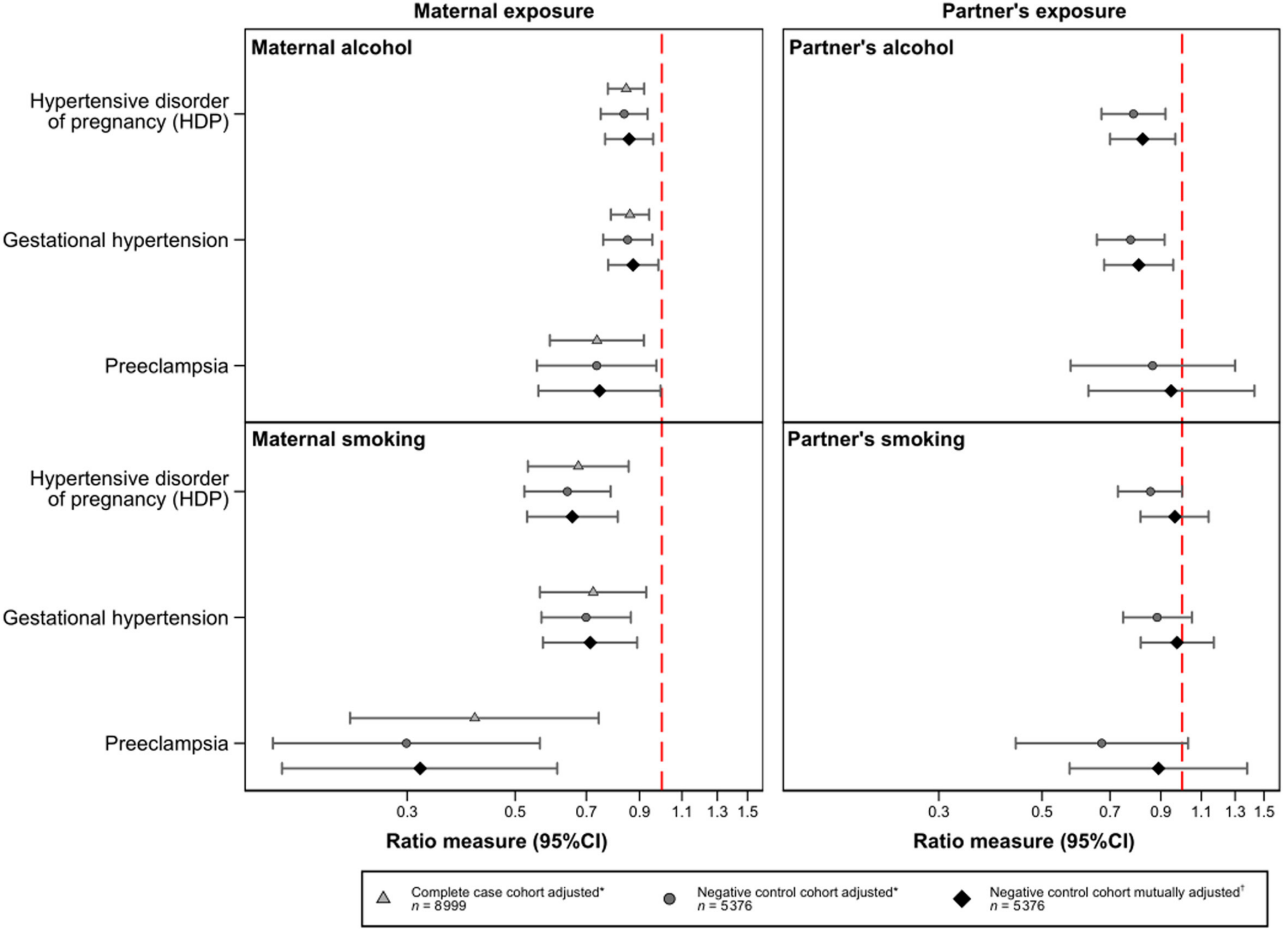
Primary and negative control analysis showing associations between maternal alcohol intake and smoking during pregnancy, as well as partner's alcohol use and smoking during pregnancy, and maternal HDP, gestational hypertension and preeclampsia. Association between alcohol and smoking during pregnancy in mothers and partners. One category increase in maternal alcohol intake (nondrinker, low to moderate or heavy drinker), is associated with a decreased odds of developing hypertensive disorder of pregnancy (HDP) in both the complete case cohort and the negative control cohort, both adjusted and mutually adjusted models (mutually adjusted odds ratio, 0.86; [95% CI, 0.77–0.96]). Similarly, partner's drinking (in the same increasing levels as described for maternal alcohol intake) is associated with a decreased odds of HDP in the adjusted and mutually adjusted model. Any maternal smoking during pregnancy (smoker or nonsmoker) shows a strong negative association with HDP in all cohorts and models, as compared with no smoking; partner's smoking during pregnancy, however, is not associated with maternal HDP risk when mutually adjusting for maternal smoking. *Adjusted for age, body mass index, smoking (in the alcohol model), alcohol (in the smoking model), parity, race or ethnicity, educational attainment, and marital status (maternal or partner covariates depending on the exposure model). ^†^Mutually adjusted for all covariates in the adjusted models plus mother/partner alcohol intake/smoking (depending on the exposure model).

Figure 2 shows the association of maternal alcohol intake during pregnancy with HDP in women with complete data (n=8999), which we refer to as the complete case cohort. The likelihood ratio test comparing model A with model B showed that the more parsimonious model A (alcohol as a 3‐level continuous variable) provided as good a fit to the data as model B (alcohol as 2 dummy variables) (*P*=0.87), thus no evidence of a nonlinear association. A 1‐category increase in alcohol intake was associated with lower odds of developing HDP (adjusted OR, 0.85; [95% CI, 0.78–0.92], *P*<0.001). Similarly, the adjusted relative risk ratio for the multinomial logistic regression was 0.86 (95% CI, 0.79–0.94, *P*=0.001) for gestational hypertension and 0.74 (95% CI, 0.59–0.92, *P*=0.007) for preeclampsia (Figure 2, Table S6).

When restricting to the sample of pregnancies with complete data on both mothers and partners (n=5376) (Figure 1), which we refer to as the negative control cohort, we obtained similar results that persisted after mutual adjustment (mutually adjusted OR, 0.86; [95% CI, 0.77–0.96], *P*=0.008) (Figure 2, Table S7).

Heavy drinkers were split into heavy nonbinge and heavy binge drinking (Data S1 and S2) to ascertain whether the protective effect may be driven by those drinking “little and often.” Characteristics of heavy nonbinge and binge drinkers were described in Table S8; both binge and nonbinge drinking were inversely associated with HDP, and CIs overlapped between drinking categories (Table S9).

### Negative Control Analysis Using Partner's Alcohol Intake

In adjusted analyses, there was evidence that partner's drinking was associated with maternal HDP risk even after mutual adjustment for maternal drinking (mutually adjusted OR, 0.82; [95% CI, 0.70–0.97], *P*=0.018, Figure 2).

An inverse association was observed with gestational hypertension; however, there was little evidence of association of partner's drinking with preeclampsia (OR, 0.95; [95% CI, 0.63–1.43], *P*=0.79, Figure 2). The number of partners who were nondrinkers was lower than the number of mothers, resulting in a smaller number of preeclamptic pregnancies in that exposure category (Figure 2, Table S10).

### Negative Control Analysis Using Smoking During Pregnancy

As shown in Figure 2, we found evidence that maternal smoking during pregnancy was strongly associated with lower HDP risk in both the complete case and negative control cohort, with results almost unchanged after adjusting for partner's smoking (mutually adjusted OR, 0.66; [95% CI, 0.53–0.81], *P*<0.001). We found similar results for gestational hypertension (OR, 0.71; [95% CI, 0.57–0.89], *P*=0.003) and a stronger association for preeclampsia (OR, 0.32; [95% CI, 0.17–0.61], *P*=0.001). On the other hand, adjustment for maternal smoking affected the estimates for partner's smoking. Based on mutually adjusted analyses, there was little evidence of association of partner's smoking with HDP, both overall and separately for gestational hypertension and preeclampsia (OR, 0.97; [95% CI, 0.81–1.14], *P*=0.682 for HDP) (Figure 2, Tables S11 through S13).

### Beverage Type Analysis

**Figure 3:**
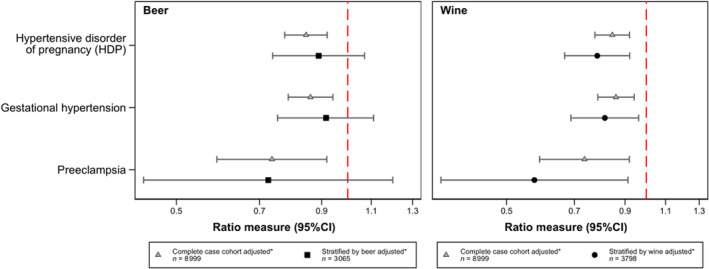
Beverage type analysis showing associations between beer and wine consumption and HDP, gestational hypertension and preeclampsia. Findings from the complete case cohort, showing the ratio measure for 1‐category increase of maternal drinking shown in both panels, adjusted for confounders. Below each finding from the complete case cohort are the results of stratifying by beverage type showing the ratio measure for 1‐category increase in beer or wine intake during pregnancy. *Adjusted for age, body mass index, before and during pregnancy smoking (binary), parity, race or ethnicity, educational attainment, and marital status. HDP indicates hypertensive disorder of pregnancy.

Beer drinkers were much more likely to smoke before and during pregnancy and less likely to be married than nondrinkers (Table S14). Those who drank wine during pregnancy were older, more likely to be White, and much more likely to have a degree than nondrinkers (Table S15). We compared risk of HDP stratified by beverage type (Figure 3). Point estimates were consistently more extreme for wine compared with beer, and the former but not the latter showed evidence of an association with lower HDP risk, although CIs overlap between these analyses (Tables S16 and S17).

To understand drinking patterns in beer and wine drinkers during pregnancy, we compared binge drinking and reported use of other alcoholic drinks (spirits/other). Beer drinkers were more likely to report binge drinking during pregnancy and although there were no differences in intake of other drinks between beer and wine drinkers, there were significantly more missing data for these questions for beer than wine drinkers (Table S18). Differing distributions of spirit intake and missing data between beer and wine demonstrate the difference in social patterning of wine and beer drinking.

### Complete Case Cohort Sensitivity Analyses

**Figure 4:**
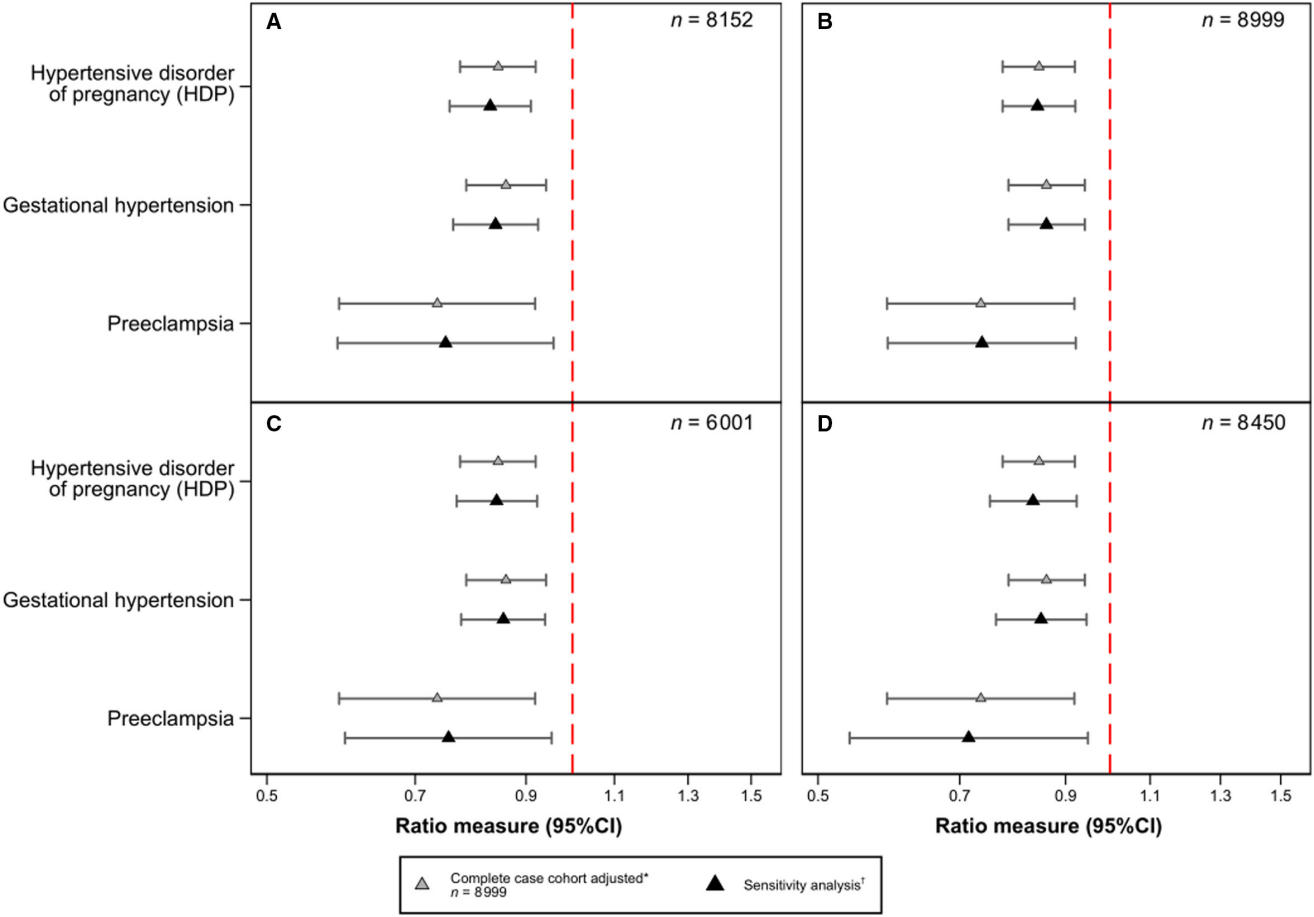
Sensitivity analyses showing associations between alcohol intake during pregnancy and HDP, gestational hypertension, and preeclampsia. **A**, Excluding those who had diabetes, kidney disease, arthritis, or multiple pregnancy. **B**, Using number of cigarettes per day (0, 1–4, 5–9, 10–14, 15–19, 20–29, and 30+). **C**, Excluding those who reported their alcohol drinking after 20 weeks' gestation. **D**, Excluding those who reported abstaining from alcohol before their pregnancy. *Adjusted for age, body mass index (BMI), before and during pregnancy smoking (binary), parity, race or ethnicity, educational attainment, and marital status. ^†^Adjusted for age, BMI, before and during pregnancy smoking (binary in model [1] and [3], categorical in model [2]), parity, race or ethnicity, educational attainment, and marital status. The denominator in each analysis is different depending on the criteria of the sensitivity analysis; for example, [1] was performed in those participants from the complete case cohort who had not reported kidney disease or arthritis during pregnancy, did not have diabetes during pregnancy, and had singleton pregnancies (n=8152). HDP indicates hypertensive disorder of pregnancy.

Figure 4 summarizes the findings from the primary analysis in the complete case cohort overlaid on each of the 4 sensitivity analysis panels for reference (Data S2). The sensitivity analyses suggested that comorbidities among those who developed HDP, differential exposure misclassification (HDP development influencing reporting of the alcohol intake during pregnancy), residual confounding by smoking, and potential poorer health of nondrinkers before pregnancy had little to no effect on our overall estimates (Tables S19 through S22).

## DISCUSSION

We found that maternal alcohol intake during pregnancy was negatively associated with any HDP, both gestational hypertension and preeclampsia, which was also confirmed in multiple sensitivity analyses. In the negative control analysis, partner's drinking was also inversely associated with maternal HDP, even after adjusting for maternal alcohol intake during pregnancy. These findings point against a causal interpretation of the maternal alcohol‐HDP association.

A (not yet peer‐reviewed) recent systematic review identified an inverse association between alcohol intake during pregnancy and preeclampsia when stratifying by prospective studies but not when including all eligible studies.^13^ In this review, only 2 of the included prospective studies used multivariable analyses to account for confounding. The first was a multicountry cohort study comparing those who quit drinking alcohol before 15 weeks' gestation with those who did not drink alcohol, finding that the former pattern of alcohol intake during pregnancy was associated with a decreased risk of preeclampsia.^10^ In the other, Iwama et al. observed HDP point estimates below 1 for those drinking almost no alcohol and less than 19 units of alcohol per week compared with none when adjusting for covariates but with large SEs and wide CIs because of small numbers in the drinking groups.^29^ The largest included retrospective study was an American record linkage analysis, which found that 1 to 2 drinks per week prenatally were negatively associated with preeclampsia compared with none in minimally adjusted models (adjusted OR, 0.82; [95% CI, 0.74–0.90]).^7^ Our findings were consistent with the results of these studies examining similar levels of alcohol intake. However, our unique take of running in parallel an analysis of partner's exposure revealed that a causal effect is highly unlikely.

The main strength of the present study is that we uniquely applied a negative control design using partner's alcohol intake during pregnancy. This approach provided a clearer insight into whether the association that was observed in the analysis of maternal alcohol intake was potentially causal, eventually concluding that shared confounding was a much more likely explanation. We additionally used smoking during pregnancy to validate this approach in the context of our data and showed that the association between partner smoking and HDP attenuated considerably when adjusting for maternal smoking. The validation step provided further support to our interpretation that shared (residual) confounding may be driving our inverse estimates of the prenatal alcohol‐HDP association.

Confounding by SEP poses an additional risk to inferring causality for the prenatal alcohol‐HDP association results. The J‐shaped curve is well discussed in alcohol and cardiovascular health epidemiology, where low‐to‐moderate amounts of alcohol intake appear to confer cardioprotective effects.^30^ Whether this is causal or a result of confounding by SEP is hotly debated. A large Mendelian randomization meta‐analysis, which is less prone to the limitations suffered by traditional observational analyses, found that those with alleles associated with lower alcohol intake had a more favorable cardiovascular profile than those without the variant, suggesting that the J‐shaped curve may be a result of confounding by SEP.^5^ Given that types of beverages consumed are also socially patterned, granular data on beer and wine intake in our cohort allowed us to run additional analyses separately for participants who drank beer and not wine (and vice versa). Investigating beer and wine separately in a beverage type analysis can be seen as an alternative method to capture some residual socioeconomic confounding that may not have been adequately accounted for by highest maternal educational attainment. The beverage type analysis showed wine to have a stronger inverse association with HDP than beer, which is consistent with the often‐reported protective effect observed for wine drinking and health outcomes.^31^ The most likely explanation for wine's protective effect on health is that wine drinkers share other characteristics that convey this benefit over nonwine drinkers, inadequately accounted for in our beverage‐type analysis and previously published studies, as opposed to a causal effect.

We were able to run a number of sensitivity analyses to address the possibility of different types of bias explaining our results. First, we excluded those who reported comorbidities associated with HDP that may have affected alcohol intake: diabetes,^21^, ^22^ kidney disease,^23^ rheumatoid arthritis,^24^ and multiple pregnancy^22^ in order to limit reverse causation (ill health causing drinking behavior, ie, abstaining from drinking). We then excluded those who reported abstaining from alcohol before their pregnancy because of potential differences in risk of the outcome between nondrinkers and drinkers before pregnancy,^32^, ^33^ again to reduce the impact of reverse causation. Given the potential for recall bias thus differential exposure misclassification, we restricted the cohort to women who had reported their drinking habits before 20 weeks' gestation (the earliest point in pregnancy that HDP can be diagnosed). The findings from these sensitivity analyses mirrored the primary analysis and suggested that behavior modification based on health and behavior reporting based on pregnancy progression were not playing a significant role in the observed association from the primary analysis. However, it remains important to consider the potential effect that discussions with health care professionals during early antenatal appointment could have on behavior or reporting of alcohol intake. Smoking has been repeatedly shown to be associated with decreased HDP risk^12^ and is correlated with alcohol use, so residual confounding by smoking behavior could introduce bias, strengthening the inverse association. Using multiple measures of smoking throughout pregnancy from multiple questionnaires, we were able to mitigate as much of the confounding by smoking as permitted by the data we have in ALSPAC.

### Strengths and Limitations

In addition to our negative control exposure analysis and multiple sensitivity analyses, a notable strength is the prospective collection of alcohol intake, which wards against recall bias. The collection of outcome data on HDP from obstetric records improved reliability and reduced amounts of missing data. This study did have some limitations. First, as disclosed in the Methods section, this study was not preregistered on Open Science Framework; however, all code for the cleaning and analysis is available on GitHub for transparency. Although we used definitions for alcohol intake and HDP that applied to the early 1990s when study pregnancies occurred, it is important to note that practice, diagnosis, and behaviors have changed over the past 3 decades. Confounding is often problematic in observational studies and residual confounding is likely. Although we did not account for physical activity^34^ and nutrition,^35^ adjustment for BMI and the beverage‐type analysis capturing unmeasured confounding by SEP were deemed sufficient in this case. Despite the large sample size, the number of women with preeclampsia was modest, though in line with other published estimates,^36^ supporting generalizability of this study. Exposure misclassification may have been an issue in this study, especially if heavy drinkers underreported their alcohol intake because of desirability bias. Although we used baseline variables in ALSPAC, thus participant attrition was relatively low, complete cases included in the analysis were less likely to drink or smoke during pregnancy, more likely to be older, married, and have higher educational attainment affecting internal validity. Participant attrition was particularly relevant for smoking during pregnancy, where those who reported smoking during pregnancy were less likely to be retained in the complete case cohort; given the correlation between alcohol intake and smoking, alcohol's association with HDP may have been underestimated.

## CONCLUSIONS

In conclusion, we found that both maternal and partner's alcohol intake during pregnancy were inversely associated with risk of any HDP, including gestational hypertension and preeclampsia. Our negative control analysis and the stronger protective effect of wine (as opposed to beer) compared with not drinking during pregnancy suggests that the association is not likely to reflect a direct, causal effect of maternal alcohol intake. These findings should be triangulated with those obtained using different methods and analytical strategies, for example, Mendelian randomization, to provide clarity on the true nature of this association.

## Sources of Funding

This research was performed in the UK Medical Research Council Integrative Epidemiology Unit (grant number: MC_UU_00011/7) and also supported by the National Institute for Health Research Bristol Biomedical Research Centre at University Hospitals Bristol National Health Service Trust and the University of Bristol. The Wellcome Trust also funds FZM's PhD studentship (grant reference: 218495/Z/19/Z) and Zuccolo was supported by a UK MRC fellowship (grant number: G0902144). Fraser was supported by an MRC personal fellowship (grant reference: MR/M009351/1). The UK Medical Research Council and the Wellcome Trust (grant reference: 217065/Z/19/Z) and the University of Bristol provide core support for ALSPAC. Further details of grant funding for ALSPAC are available on their website.

## Disclosures

None.

## Supporting information

Data S1–S2
Tables S1–S22
References 37, 38
