# Supplementary material for: Alcohol Intake and Hypertensive Disorders of Pregnancy: A Negative Control Analysis in the ALSPAC Cohort
Source: J Am Heart Assoc. 2022 Sep 29;11(19):e025102. doi: 10.1161/JAHA.121.025102 (PMC9673719; doi:10.1161/JAHA.121.025102)
Supplement: Supplementary file 1 — Data S1–S2 Tables S1–S22 References 37, 38 [file JAH3-11-e025102-s001.pdf]

SUPPLEMENTAL MATERIAL

Data S1. Stratifying heavy drinking by binge drinking

As described in the methods section, the heavy drinking category was further stratified by binge and non-binge drinkers. It has been shown that different forms of drinking are more frequent in different socioeconomic groups: those who drink little and often (for example, one glass of wine every night) and those who drink heavily once a week, are both heavy drinkers in our analysis. However, they have been shown in this sample (Table S5) to be characteristically different from one another; we deduced that this may have impacted on their underlying risk of outcome. There was a slight attenuation of the protective effect for heavy binge drinkers vs heavy non-binge drinkers when comparing with non-drinkers (as per the primary analysis), but the direction of effect was the same for all three outcomes and the intervals did not include one (Table S6).

Data S2. Sensitivity analysis findings

Excluding participants with higher risk of HDP (diabetes, kidney disease or arthritis during pregnancy and non-singleton pregnancies)

It has been shown that those who have diabetes, kidney disease, arthritis or non-singleton pregnancies have a higher risk of developing HDP. With the exchangeability in question between this group and those who did not experience any of these pregnancy complications, we performed a sensitivity analysis by which we excluded those women who had reported or were diagnosed with any of these risk factors for HDP. Other than slightly attenuating the effect, probably due to decreased power following their exclusion, both the crude and adjusted models concluded a reduction in relative risk, following alcohol intake in pregnancy, of both gestational hypertension and preeclampsia (adjusted relative risk ratio 0.84, 95% confidence interval 0.76 to 0.93, P-value<0.001 and 0.75, 0.59 to 0.96, P=0.021, respectively) (part (i), Figure 4).

Stratifying smoking during pregnancy

It has been shown on multiple occasions that smoking during pregnancy is associated with a protective effect for preeclampsia (37, 38). We also observed this in ALSPAC, using smoking during pregnancy and risk of HDP to corroborate our findings for the partner's alcohol negative control analysis (Figure 2). Given that smoking is associated with drinking alcohol during pregnancy it was important to reduce any residual confounding not accounted for by using a binary smoking covariate. Having categorised smoking during pregnancy into average number per day (0, 1–4, 5–9, 10–14, 15–19, 20–29 and 30+), we observed no difference in effect from the model that adjusted for binary smoking during pregnancy (any or none) (adjusted odds ratio 0.85, 95% confidence interval 0.78 to 0.92, P-value<0.001) (part (ii), Figure 4).

Excluding respondent's post-20 weeks' gestation

Given the diagnosis of gestational hypertension or preeclampsia occurs at 20 weeks' gestation or later, we considered the potential for knowledge of the outcome to have influenced reporting of the exposure, had the questionnaire been filled out after a diagnosis could have been made. With this in mind, we restricted the analysis to those who had filled in the questionnaire prior to 20 weeks' gestation (thus excluding responses from the postpartum questionnaire regarding drinking habits in the last two months of pregnancy) (part (i) Figure 4). The protective effect persisted in both the logistic and multinomial models having restricted to participants responding prior to 20 weeks' gestation (adjusted relative risk ratio 0.85, 95% confidence interval 0.76 to 0.95, P-value=0.003 and 0.72, 0.54 to 0.95, P=0.020, for gestational hypertension and preeclampsia, respectively).

Excluding abstainers prior to pregnancy

It has been shown that those who abstain from alcohol outside of pregnancy are characteristically different and have exhibited different risks of morbidity and mortality compared with their drinking counterparts (32, 33). With the exchangeability in question between this group and those who did not abstain before pregnancy and their risk of the outcome, we performed a sensitivity analysis by which we excluded those women who had reported to have abstained from alcohol prior to pregnancy. Other than slightly attenuating the effect, probably due to decreased power following their exclusion, both the crude and adjusted models concluded a reduction in relative risk, following alcohol intake in pregnancy, of both gestational hypertension and preeclampsia (adjusted relative risk ratio 0.86, 95% confidence interval 0.78 to 0.95, P-value=0.001 and 0.76, 0.60 to 0.95, P=0.019, respectively) (part (iv), Figure 4).

Table S1. Summary of the variables in the full ALSPAC cohort used in the complete case (primary) analysis (*n*=15,442), those in the complete case cohort (*n*=8,999) and excluded, incomplete cases for exposure, outcomes, and covariates.

Characteristic	Categories	Available data (n = 15,442) *n* (%)	Categorical data (n = 15,442) *n* (%)	Complete records (n = 8,999) *n* (%)	Records with incomplete data *n*	Excluded *n* (%)
Maternal alcohol use in pregnancy	Non-drinker	12,373 (80)	3,194 (26)	2,415 (27)	3,030	679 (22)
	Low-to-moderate		6,439 (52)	4,696 (52)		1,569 (52)
	Heavy		2,740 (22)	1,888 (21)		782 (26)
Maternal age	Under 25	13,897 (90)	3,337 (24)	1,719 (19)	4,554	1,563 (34)
	25 and over		10,560 (76)	7,280 (81)		2,991 (66)
Maternal body mass index (BMI)	Underweight	11,524 (75)	577 (5)	425 (5)	2,181	141 (7)
	Normal		8,562 (74)	6,767 (75)		1,572 (73)
	Overweight		1,733 (15)	1,355 (15)		316 (15)
	Obese		652 (6)	452 (5)		152 (6)
Maternal smoking before pregnancy	Non-smoker	13,193 (85)	8,723 (66)	6,121 (68)	3,850	2,344 (61)
	Smoker		4,470 (34)	2,878 (32)		1,506 (39)
Maternal smoking during pregnancy	Non-smoker	11,994 (78)	8,309 (69)	6,703 (75)	2,651	1,330 (50)
	Smoker		3,685 (31)	2,296 (25)		1,321 (50)
Maternal parity	Nulliparous	12,960 (84)	5,804 (45)	4,006 (45)	3,617	1,614 (45)
	Multiparous		7,156 (55)	4,993 (55)		2,003 (55)
Maternal ethnicity	White	12,251 (79)	11,909 (97)	8,834 (98)	2,908	2,735 (94)
	Non-white		342 (3)	165 (2)		173 (6)
Maternal educational attainment	A levels or less	12,321 (80)	10,736 (87)	7,754 (86)	2,978	2,687 (90)
	Degree		1,585 (13)	1,245 (14)		291 (10)
Maternal marital status	Not currently married	13,548 (88)	3,523 (26)	2,000 (22)	4,205	1,448 (34)
	Married		10,025 (74)	6,999 (78)		2,762 (66)
Maternal HDP	Normotensive	13,681 (89)	11,447 (84)	7,509 (83)	4,338	3,594 (83)
	Gestational hypertension		1,937 (14)	1,308 (15)		629 (15)
	Preeclampsia		297 (2)	182 (2)		115 (3)

Table S2. Summary of the variables in the full ALSPAC cohort used in the negative control analysis (*n*=15,442), those in the negative control cohort (*n*=5,376) and excluded, incomplete cases for exposure, outcomes, and covariates.

Characteristic	Categories	Available data (n = 15,442) *n* (%)	Categorical data (n = 15,442) *n* (%)	Complete records (n = 5,376) *n* (%)	Records with incomplete data *n*	Excluded *n* (%)
Partner alcohol intake in pregnancy	Non-drinker	10,380 (67)	268 (3)	141 (3)	3,885	82 (2)
	Low-to-moderate		7,594 (73)	3,943 (73)		2,839 (73)
	Heavy		2,518 (24)	1,292 (24)		964 (25)
Partner age	Under 25	8,146 (53)	941 (12)	527 (10)	1,597	280 (18)
	25 and over		7,205 (88)	4,849 (90)		1,317 (82)
Partner body mass index (BMI)	Underweight	12,451 (81)	1,504 (12)	247 (5)	5,915	801 (14)
	Normal		8,562 (69)	4,049 (75)		3,979 (67)
	Overweight		1,733 (14)	816 (15)		816 (14)
	Obese		652 (5)	264 (5)		319 (5)
Partner smoking during pregnancy	Non-smoker	9,418 (61)	5,837 (62)	3,561 (66)	2,869	1,551 (54)
	Smoker		3,581 (38)	1,815 (34)		1,318 (46)
Partner parity	Nulliparous	12,960 (84)	5,804 (45)	2,580 (48)	6,411	2,646 (41)
	Multiparous		7,156 (55)	2,796 (52)		3,765 (59)
Partner ethnicity	White	9,746 (63)	9,459 (97)	5,291 (98)	3,197	3,037 (95)
	Non-white		287 (3)	85 (2)		160 (5)
Partner educational attainment	A levels or less	9,803 (64)	7,892 (81)	4,145 (77)	3,254	2,808 (86)
	Degree		1,911 (19)	1,231 (23)		446 (14)
Partner marital status	Not currently married	13,548 (88)	3,523 (26)	950 (18)	6,999	2,321 (33)
	Married		10,025 (74)	4,426 (82)		4,678 (67)
Maternal HDP	Normotensive	13,681 (89)	11,447 (84)	4,420 (82)	7,139	6,043 (85)
	Gestational hypertension		1,937 (14)	837 (16)		938 (13)
	Preeclampsia		297 (2)	119 (2)		158 (2)

Table S3. Predictors of being a complete case in available data for each maternal variable

Characteristic	Category	Crude OR (95%CI)
Maternal alcohol intake in pregnancy	Non-drinker	1.00 (referent)
	Low-to-moderate	0.84 (0.76 to 0.93)
	Heavy	0.70 (0.62 to 0.78)
Maternal age	Under 25	1.00 (referent)
	25 and over	2.24 (2.07 to 2.43)
Maternal body mass index (BMI)	Underweight	1.00 (referent)
	Normal	1.44 (1.18 to 1.75)
	Overweight	1.46 (1.15 to 1.81)
	Obese	1.23 (0.94 to 1.62)
Maternal smoking before pregnancy	Non-smoker	1.00 (referent)
	Smoker	0.73 (0.68 to 0.79)
Maternal smoking during pregnancy	Non-smoker	1.00 (referent)
	Smoker	0.34 (0.31 to 0.37)
Maternal parity	Nulliparous	1.00 (referent)
	Multiparous	1.00 (0.93 to 1.08)
Maternal ethnicity	White	1.00 (referent)
	Non-white	0.33 (0.27 to 0.41)
Maternal educational attainment	A-levels or lower	1.00 (referent)
	University degree	1.48 (1.29 to 1.69)
Maternal marital status	Not currently married	1.00 (referent)
	Married	1.84 (1.70 to 1.99)
Maternal HDP	Normotensive	1.00 (referent)
	HDP	0.92 (0.83 to 1.01)

Table S4. Predictors of being a complete case in available data for each partner variable

Characteristic	Category	Crude OR (95%CI)
Partner alcohol intake in pregnancy	Non-drinker	1.00 (referent)
	Low-to-moderate	0.74 (0.57 to 0.96)
	Heavy	0.71 (0.54 to 0.93)
Partner age	Under 25	1.00 (referent)
	25 and over	1.89 (1.63 to 2.21)
Partner body mass index (BMI)	Underweight	1.00 (referent)
	Normal	1.31 (1.18 to 1.46)
	Overweight	1.28 (1.12 to 1.47)
	Obese	1.19 (0.99 to 1.43)
Partner smoking during pregnancy	Non-smoker	1.00 (referent)
	Smoker	0.62 (0.57 to 0.68)
Partner parity	Nulliparous	1.00 (referent)
	Multiparous	0.76 (0.70 to 0.81)
Partner ethnicity	White	1.00 (referent)
	Non-white	0.38 (0.30 to 0.48)
Partner educational attainment	A-levels or lower	1.00 (referent)
	University degree	1.81 (1.62 to 2.04)
Partner marital status	Not currently married	1.00 (referent)
	Married	2.21 (2.04 to 2.39)
Maternal HDP	Normotensive	1.00 (referent)
	HDP	1.16 (1.06 to 1.27)

Table S5. Characteristics of normotensive participants (*n*=7,509) compared to participants with HDP (*n*=1,490)

	Normotensive	Hypertensive disorder of pregnancy
	7,509	1,490
Age at delivery		
Mean, years (SD*)	28.6 (4.7)	28.3 (4.9)
BMI† pre-pregnancy		
Mean, kg/m^2 (SD*)	22.5 (3.3)	24.7 (4.9)
Smoking		
Any pre-pregnancy, *n* (%)	2,456 (32.7)	422 (28.3)
Any during pregnancy, *n* (%)	1,995 (26.6)	301 (20.2)
Parity (18 weeks' gestation)		
Multiparous, *n* (%)	4,384 (58.4)	609 (40.9)
Ethnicity (32 weeks' gestation)		
Non-white, *n* (%)	146 (1.9)	19 (1.3)
Educational attainment (32 weeks' gestation)		
University degree, *n* (%)	1,040 (13.9)	205 (13.8)
Marital status		
Married, *n* (%)	5,839 (77.8)	1,160 (77.9)

* Standard deviation
† Body mass index

Table S6. Maternal alcohol intake during pregnancy and HDP in complete case cohort (*n*=8,999)

Maternal outcome	Maternal alcohol intake during pregnancy			Unadjusted model		Adjusted model	
	Heavy *n* (%)	Low-to-moderate *n* (%)	None *n* (%)	OR* (95%CI)	*p*-value	OR*, † (95%CI)	*p*-value
HDP – *n* complete cases	1,888	4,696	2,415	-	-	-	-
Yes	259 (13.7)	765 (16.3)	466 (19.3)	0.82 (0.75 to 0.88)	<0.001	0.85 (0.78 to 0.92)	<0.001
				RR‡ (95%CI)	*p*-value	RR†, ‡ (95%CI)	*p*-value
Gestational hypertension – *n* complete cases	1,888	4,696	2,415	-	-	-	-
Yes	229 (12.1)	681 (14.5)	398 (16.5)	0.83 (0.76 to 0.91)	<0.001	0.86 (0.79 to 0.94)	0.001
Preeclampsia – *n* complete cases	1,888	4,696	2,415	-	-	-	-
Yes	30 (1.6)	84 (1.8)	68 (2.8)	0.70 (0.56 to 0.87)	0.001	0.74 (0.59 to 0.92)	0.007

* Odds ratio generated using logistic regression

† Adjusted for covariates: maternal age at delivery, maternal body mass index (pre-pregnancy), pre-pregnancy smoking (binary), smoking during pregnancy (binary), parity (0, 1, 2 or ≥3), maternal ethnicity (white or non-white), maternal education (32 weeks' gestation) and marital status

‡ Relative risk ratio generated by multinomial logistic regression

Table S7. Maternal alcohol intake during pregnancy and HDP in negative control cohort (*n*=5,376)

Maternal outcome	Maternal alcohol intake during pregnancy			Unadjusted model		Adjusted model		Mutually adjusted model	
	Heavy *n* (%)	Low-to-moderate *n* (%)	None *n* (%)	OR* (95%CI)	*p*-value	OR*, † (95%CI)	*p*-value	OR*, †, § (95%CI)	*p*-value
HDP – *n* complete cases	1,030	2,872	1,474	-	-	-	-	-	-
Yes	153 (14.9)	493 (17.2)	310 (21.0)	0.80 (0.72 to 0.89)	<0.001	0.84 (0.75 to 0.94)	0.002	0.86 (0.77 to 0.96)	0.008
				RR‡ (95%CI)	*p*-value	RR†, ‡ (95%CI)	*p*-value	RR†, ‡, § (95%CI)	*p*-value
Gestational hypertension – *n* complete cases	1,030	2,872	1,474	-	-	-	-	-	-
Yes	137 (13.3)	434 (15.1)	266 (18.1)	0.82 (0.74 to 0.92)	<0.001	0.85 (0.76 to 0.96)	0.007	0.87 (0.78 to 0.98)	0.026
Preeclampsia – *n* complete cases	1,030	2,872	1,474	-	-	-	-	-	-
Yes	16 (1.6)	59 (2.1)	44 (3.0)	0.69 (0.52 to 0.90)	0.007	0.74 (0.55 to 0.98)	0.033	0.75 (0.56 to 0.99)	0.045

* Odds ratio generated using logistic regression
† Adjusted for covariates: maternal age at delivery, maternal body mass index (pre-pregnancy), pre-pregnancy smoking (binary), smoking during pregnancy (binary), parity (0, 1, 2 or ≥3), maternal ethnicity (white or non-white), maternal education (32 weeks' gestation) and marital status
‡ Relative risk ratio generated by multinomial logistic regression
§ Mutually adjusted for covariates plus partner's alcohol intake during pregnancy

Table S8. Characteristics of participants by categories of alcohol intake during pregnancy, heavy stratified by binge and non-binge

	None	Low-to-moderate	Heavy non-binge	Heavy binge
	2,415	4,696	348	1,540
Age at delivery				
Mean, years (SD*)	27.7 (4.7)	28.9 (4.6)	30.4 (4.7)	28.4 (4.9)
BMI† (pre-pregnancy)				
Mean, kg/m^2 (SD*)	23.0 (4.0)	22.7 (3.6)	22.5 (3.3)	23.2 (3.0)
Smoking				
Any pre-pregnancy, *n* (%)	705 (29.2)	1,299 (27.7)	117 (33.6)	757 (49.2)
Any during pregnancy, *n* (%)	552 (22.9)	991 (21.1)	91 (26.2)	662 (43.0)
Parity (18 weeks' gestation)				
Multiparous, *n* (%)	1,280 (53.0)	2,589 (55.1)	199 (57.2)	925 (60.1)
Ethnicity (32 weeks' gestation)				
Non-white, *n* (%)	61 (2.5)	80 (1.7)	4 (1.2)	20 (1.3)
Educational attainment (32 weeks' gestation)				
University degree, *n* (%)	232 (9.6)	792 (16.9)	102 (29.3)	119 (7.7)
Marital status				
Married, *n* (%)	1,904 (78.8)	3,792 (80.8)	273 (78.5)	1,030 (66.9)

* Standard deviation

† Body mass index

Table S9. Maternal alcohol intake during pregnancy and HDP, expanding heavy drinking to binge and non-binge (n=8,999), including alcohol exposure as a categorical exposure

		Unadjusted model		Adjusted model	
Type of drinking	Maternal outcome	OR[*] (95%CI)	*p*-value	OR[*, †] (95%CI)	*p*-value
	HDP *n* (%)	-	-	-	-
None	466/2,415 (19.3)	1.00 (reference)	-	1.00 (reference)	-
Low-to-moderate	765/4,696 (16.3)	0.81 (0.72 to 0.92)	0.002	0.85 (0.74 to 0.97)	0.019
Heavy non-binge	43/348 (12.4)	0.59 (0.42 to 0.82)	0.002	0.64 (0.45 to 0.90)	0.012
Heavy binge	216/1,540 (14.0)	0.68 (0.57 to 0.81)	<0.001	0.73 (0.60 to 0.88)	0.001
		RR[‡] (95%CI)	*p*-value	RR[†, ‡] (95%CI)	*p*-value
	Gestational hypertension				
None	398/2,415 (16.5)	1.00 (reference)	-	1.00 (reference)	-
Low-to-moderate	681/4,696 (14.5)	0.85 (0.74 to 0.97)	0.017	0.88 (0.77 to 1.02)	0.083
Heavy non-binge	36/348 (10.3)	0.58 (0.40 to 0.83)	0.003	0.62 (0.43 to 0.90)	0.013
Heavy binge	193/1,540 (12.5)	0.71 (0.59 to 0.86)	<0.001	0.76 (0.63 to 0.92)	0.006
	Preeclampsia	-	-	-	-
None	68/2,415 (2.8)	1.00 (reference)	-	1.00 (reference)	-
Low-to-moderate	84/4,696 (1.8)	0.61 (0.44 to 0.85)	0.003	0.66 (0.48 to 0.93)	0.017
Heavy non-binge	7/348 (2.0)	0.66 (0.30 to 1.45)	0.297	0.74 (0.33 to 1.66)	0.463
Heavy binge	23/1,540 (1.5)	0.50 (0.31 to 0.80)	0.004	0.54 (0.33 to 0.87)	0.013

[*] Odds ratio generated using logistic regression
[†] Adjusted for covariates: maternal age at delivery, maternal body mass index (pre-pregnancy), pre-pregnancy smoking (binary), smoking during pregnancy (binary), parity (0, 1, 2 or ≥3), maternal ethnicity (white or non-white), maternal education (32 weeks' gestation) and marital status
[‡] Relative risk ratio generated by multinomial logistic regression

Table S10. Partner's alcohol intake during pregnancy and maternal HDP in negative control cohort (*n*=5,376)

Maternal outcome	Partner's alcohol intake during pregnancy			Unadjusted model		Adjusted model		Mutually adjusted model	
	Heavy *n* (%)	Low-to-moderate *n* (%)	None *n* (%)	OR* (95%CI)	*p*-value	OR*, † (95%CI)	*p*-value	OR*, †, § (95%CI)	*p*-value
HDP – *n* complete cases	1,292	3,943	141	-	-	-	-	-	-
Yes	196 (15.2)	728 (18.5)	32 (22.7)	0.79 (0.68 to 0.92)	0.002	0.79 (0.67 to 0.92)	0.003	0.82 (0.70 to 0.97)	0.018
				RR‡ (95%CI)	*p*-value	RR†, ‡ (95%CI)	*p*-value	RR†, ‡, § (95%CI)	*p*-value
Gestational hypertension – *n* complete cases	1,292	3,943	141	-	-	-	-	-	-
Yes	168 (13.0)	642 (16.3)	27 (19.2)	0.78 (0.66 to 0.91)	0.002	0.78 (0.66 to 0.92)	0.003	0.81 (0.68 to 0.96)	0.014
Preeclampsia – *n* complete cases	1,292	3,943	141	-	-	-	-	-	-
Yes	28 (2.2)	86 (2.2)	5 (3.6)	0.87 (0.59 to 1.29)	0.494	0.86 (0.58 to 1.30)	0.479	0.95 (0.63 to 1.43)	0.794

* Odds ratio generated using logistic regression
† Adjusted for covariates: partner's age at delivery, partner's body mass index (pre-pregnancy), pre-pregnancy smoking (binary), smoking during pregnancy (binary), parity (0, 1, 2 or ≥3), partner's ethnicity (white or non-white), partner's education (32 weeks' gestation) and marital status
‡ Relative risk ratio generated by multinomial logistic regression
§ Mutually adjusted for covariates plus maternal alcohol intake during pregnancy

Table S11. Maternal smoking during pregnancy and HDP in complete case cohort (*n*=8,999)

Maternal outcome	Maternal smoking during pregnancy		Unadjusted model		Adjusted model	
	Any *n* (%)	None *n* (%)	OR* (95%CI)	*p*-value	OR*, † (95%CI)	*p*-value
HDP – *n* complete cases	**2,296**	**6,703**	-	-	-	-
Yes	301 (13.1)	1,189 (17.7)	0.70 (0.61 to 0.80)	<0.001	0.67 (0.53 to 0.86)	0.001
			RR‡ (95%CI)	*p*-value	RR†, ‡ (95%CI)	*p*-value
Gestational hypertension – *n* complete cases	**2,296**	**6,703**	-	-	-	-
Yes	271 (11.8)	1,037 (15.5)	0.72 (0.63 to 0.83)	<0.001	0.72 (0.56 to 0.93)	0.011
Preeclampsia – *n* complete cases	**2,296**	**6,703**	-	-	-	-
Yes	30 (1.3)	152 (2.3)	0.55 (0.37 to 0.81)	0.003	0.41 (0.23 to 0.74)	0.003

* Odds ratio generated using logistic regression

† Adjusted for covariates: maternal age at delivery, maternal body mass index (pre-pregnancy), pre-pregnancy smoking (binary), smoking during pregnancy (binary), parity (0, 1, 2 or ≥3), maternal ethnicity (white or non-white), maternal education (32 weeks' gestation) and marital status

‡ Relative risk ratio generated by multinomial logistic regression

Table S12. Maternal smoking during pregnancy and HDP in negative control cohort (*n*=5,376)

Maternal outcome	Maternal smoking during pregnancy		Unadjusted model		Adjusted model		Mutually adjusted model	
	Any *n* (%)	None *n* (%)	OR* (95%CI)	*p*-value	OR*, † (95%CI)	*p*-value	OR*, †, § (95%CI)	*p*-value
HDP – *n* complete cases	1,169	4,207	-	-	-	-	-	-
Yes	159 (13.6)	797 (18.9)	0.67 (0.56 to 0.81)	<0.001	0.64 (0.52 to 0.79)	<0.001	0.66 (0.53 to 0.81)	<0.001
			RR‡ (95%CI)	*p*-value	RR†, ‡ (95%CI)	*p*-value	RR†, ‡, § (95%CI)	*p*-value
Gestational hypertension – *n* complete cases	1,169	4,207	-	-	-	-	-	-
Yes	147 (12.6)	690 (16.4)	0.72 (0.59 to 0.87)	0.001	0.70 (0.57 to 0.86)	0.001	0.71 (0.57 to 0.89)	0.003
Preeclampsia – *n* complete cases	1,169	4,207	-	-	-	-	-	-
Yes	12 (1.0)	107 (2.5)	0.38 (0.21 to 0.69)	0.002	0.30 (0.16 to 0.56)	<0.001	0.32 (0.17 to 0.61)	0.001

* Odds ratio generated using logistic regression

† Adjusted for covariates: maternal age at delivery, maternal body mass index (pre-pregnancy), pre-pregnancy smoking (binary), alcohol intake during pregnancy (none, low-to-moderate or heavy), parity (0, 1, 2 or ≥3), maternal ethnicity (white or non-white), maternal education (32 weeks' gestation) and marital status

‡ Relative risk ratio generated by multinomial logistic regression

§ Mutually adjusted for covariates plus partner's smoking during pregnancy

Table S13. Partner's smoking during pregnancy and maternal HDP in negative control cohort (*n*=5,376)

Maternal outcome	Partner's smoking during pregnancy		Unadjusted model		Adjusted model		Mutually adjusted model	
	Any *n* (%)	None *n* (%)	OR[*] (95%CI)	*p*-value	OR[*, †] (95%CI)	*p*-value	OR[*, †, §] (95%CI)	*p*-value
HDP – *n* complete cases	1,815	3,561	-	-	-	-	-	-
Yes	298 (16.4)	658 (18.5)	0.87 (0.75 to 1.01)	0.062	0.86 (0.73 to 1.00)	0.056	0.97 (0.81 to 1.14)	0.682
			RR[‡] (95%CI)	*p*-value	RR[†, ‡] (95%CI)	*p*-value	RR[†, ‡, §] (95%CI)	*p*-value
Gestational hypertension – *n* complete cases	1,815	3,561	-	-	-	-	-	-
Yes	264 (14.6)	573 (16.1)	0.88 (0.75 to 1.03)	0.119	0.884 (0.75 to 1.05)	0.152	0.98 (0.82 to 1.17)	0.779
Preeclampsia – *n* complete cases	1,815	3,561	-	-	-	-	-	-
Yes	34 (1.9)	85 (2.4)	0.77 (0.51 to 1.14)	0.193	0.67 (0.44 to 1.03)	0.067	0.89 (0.57 to 1.38)	0.606

[*] Odds ratio generated using logistic regression
[†] Adjusted for covariates: partner's age at delivery, partner's body mass index (pre-pregnancy), pre-pregnancy smoking (binary), partner's alcohol intake during pregnancy (none, low-to-moderate or heavy), parity (0, 1, 2 or ≥3), partner's ethnicity (white or non-white), partner's education (32 weeks' gestation) and marital status
[‡] Relative risk ratio generated by multinomial logistic regression
[§] Mutually adjusted for covariates plus maternal smoking during pregnancy

Table S14. Characteristics of participants stratified by categories of beer intake, excluding those who report any wine intake.

	No alcohol in pregnancy	Low-to-moderate beer intake in pregnancy	Heavy beer intake in pregnancy
	2,415	458	192
Age at delivery			
Mean, years (SD*)	27.7 (4.68)	27.6 (4.70)	28.6 (5.1)
BMI† (pre-pregnancy)			
Mean, kg/m^2 (SD*)	23.0 (4.02)	23.0 (4.0)	22.8 (3.1)
Smoking			
Any pre-pregnancy, *n* (%)	705 (29.2)	220 (48.0)	111 (57.8)
Any during pregnancy, *n* (%)	552 (22.9)	191 (41.7)	102 (53.1)
Parity (18 weeks' gestation)			
Multiparous, *n* (%)	1,280 (53.0)	240 (52.4)	128 (66.7)
Ethnicity (32 weeks' gestation)			
Non-white, *n* (%)	61 (2.5)	3 (0.7)	6 (3.1)
Educational attainment (32 weeks' gestation)			
University degree, *n* (%)	232 (9.6)	38 (8.3)	16 (8.3)
Marital status			
Married, *n* (%)	1,904 (78.8)	317 (69.2)	110 (57.3)

* Standard deviation
† Body mass index

Table S15. Characteristics of participants stratified by categories of wine intake, excluding those who report any beer intake.

	No alcohol in pregnancy	Low-to-moderate wine intake in pregnancy	Heavy wine intake in pregnancy
	2,415	1,152	231
Age at delivery			
Mean, years (SD*)	27.7 (4.68)	29.7 (4.3)	30.9 (4.6)
BMI† (pre-pregnancy)			
Mean, kg/m² (SD*)	23.0 (4.02)	22.8 (3.4)	22.6 (3.2)
Smoking			
Any pre-pregnancy, *n* (%)	705 (29.2)	300 (26.0)	81 (35.1)
Any during pregnancy, *n* (%)	552 (22.9)	215 (18.7)	62 (26.8)
Parity (18 weeks' gestation)			
Multiparous, *n* (%)	1,280 (53.0)	642 (55.7)	147 (63.6)
Ethnicity (32 weeks' gestation)			
Non-white, *n* (%)	61 (2.5)	14 (1.2)	1 (0.4)
Educational attainment (32 weeks' gestation)			
University degree, *n* (%)	232 (9.6)	235 (20.4)	58 (25.1)
Marital status			
Married, *n* (%)	1,904 (78.8)	979 (85.0)	192 (83.1)

* Standard deviation

† Body mass index

Table S16. Maternal beer intake during pregnancies and HDP restricted to those with beer and wine data available (*n*=3,065)

Maternal outcome	Maternal beer drinking			Unadjusted model		Adjusted model	
	Heavy *n* (%)	Low-to-moderate *n* (%)	None *n* (%)	OR* (95%CI)	*p*-value	OR*, † (95%CI)	*p*-value
HDP – *n* complete cases	**192**	**458**	**2,415**	-	-	-	-
Yes	27 (14.1)	72 (15.7)	466 (19.3)	0.81 (0.68 to 0.96)	0.017	0.89 (0.74 to 1.07)	0.217
				RR‡ (95%CI)	*p*-value	RR†, ‡ (95%CI)	*p*-value
Gestational hypertension – *n* complete cases	**192**	**458**	**2,415**	-	-	-	-
Yes	24 (12.5)	64 (14.0)	398 (16.5)	0.83 (0.69 to 1.00)	0.049	0.92 (0.75 to 1.11)	0.378
Preeclampsia – *n* complete cases	**192**	**458**	**2,415**	-	-	-	-
Yes	3 (1.6)	8 (1.8)	68 (2.8)	0.67 (0.41 to 1.08)	0.102	0.73 (0.44 to 1.20)	0.211

* Odds ratio generated using logistic regression

† Adjusted for covariates: maternal age at delivery, maternal body mass index (pre-pregnancy), pre-pregnancy smoking (binary), smoking during pregnancy (binary), parity (0, 1, 2 or ≥3), maternal ethnicity (white or non-white), maternal education (32 weeks' gestation) and marital status

‡ Relative risk ratio generated by multinomial logistic regression

Table S17. Maternal wine intake during pregnancies and HDP restricted to those with beer and wine data available (*n*=3,065)

Maternal outcome	Maternal wine drinking			Unadjusted model		Adjusted model	
	Heavy *n* (%)	Low-to-moderate *n* (%)	None *n* (%)	OR* (95%CI)	*p*-value	OR*, † (95%CI)	*p*-value
HDP – *n* complete cases	**231**	**1,152**	**2,415**	-	-	-	-
Yes	29 (12.6)	170 (14.8)	466 (19.3)	0.75 (0.64 to 0.87)	<0.001	0.78 (0.67 to 0.92)	0.003
				RR‡ (95%CI)	*p*-value	RR†, ‡ (95%CI)	*p*-value
Gestational hypertension – *n* complete cases	**231**	**1,152**	**2,415**	-	-	-	-
Yes	27 (11.7)	152 (13.2)	398 (16.5)	0.78 (0.67 to 0.91)	0.002	0.81 (0.69 to 0.96)	0.378
Preeclampsia – *n* complete cases	**231**	**1,152**	**2,415**	-	-	-	-
Yes	2 (0.9)	18 (1.6)	68 (2.8)	0.53 (0.34 to 0.82)	0.004	0.57 (0.36 to 0.91)	0.019

* Odds ratio generated using logistic regression

† Adjusted for covariates: maternal age at delivery, maternal body mass index (pre-pregnancy), pre-pregnancy smoking (binary), smoking during pregnancy (binary), parity (0, 1, 2 or ≥3), maternal ethnicity (white or non-white), maternal education (32 weeks' gestation) and marital status

‡ Relative risk ratio generated by multinomial logistic regression

Table S18. Binge drinking and intake of additional forms of alcohol in beer (*n*=650) and wine (*n*=1,383) drinking groups.

	Wine drinkers in pregnancy	Beer drinkers in pregnancy
	1,383	650
Bingeing*		
Yes, *n* (%)	380 (27.5)	293 (45.1)
Missing, *n* (%)	10 (0.7)	7 (1.1)
Other alcohol intake†		
Yes, *n* (%)	67 (4.8)	28 (4.3)
Missing, *n* (%)	229 (16.6)	258 (39.7)

* Bingeing defined as drinking 4 or more drinks in one sitting
† Other alcohol intake defined as reporting intake of spirits or “other” alcohol during pregnancy

Table S19. Maternal alcohol intake and HDP excluding those who have reported diabetes, kidney disease or arthritis during pregnancy or a non-singleton pregnancy (*n*=8,152) – (i) on Figure 4.

Maternal outcome	Maternal alcohol intake during pregnancy			Unadjusted model		Adjusted model	
	Heavy *n* (%)	Low-to-moderate *n* (%)	None *n* (%)	OR* (95%CI)	*p*-value	OR*, † (95%CI)	*p*-value
**HDP** – *n* complete cases	**1,668**	**4,294**	**1,668**	-	-	-	-
Yes	212 (12.7)	693 (16.1)	411 (18.8)	0.80 (0.73 to 0.87)	<0.001	0.83 (0.76 to 0.91)	<0.001
				RR‡ (95%CI)	*p*-value	RR†, ‡ (95%CI)	*p*-value
**Gestational hypertension** – *n* complete cases	**1,668**	**4,294**	**1,668**	-	-	-	-
Yes	188 (11.3)	621 (14.5)	357 (16.3)	0.81 (0.74 to 0.89)	<0.001	0.84 (0.76 to 0.93)	<0.001
**Preeclampsia** – *n* complete cases	**1,668**	**4,294**	**1,668**	-	-	-	-
Yes	24 (1.4)	72 (1.7)	54 (2.5)	0.72 (0.57 to 0.91)	0.007	0.75 (0.59 to 0.96)	0.021

* Odds ratio generated using logistic regression

† Adjusted for covariates: maternal age at delivery, maternal body mass index (pre-pregnancy), pre-pregnancy smoking (binary), smoking during pregnancy (binary), parity (0, 1, 2 or ≥3), maternal ethnicity (white or non-white), maternal education (32 weeks' gestation) and marital status

‡ Relative risk ratio generated by multinomial logistic regression

Table S20. Maternal alcohol intake during pregnancy and HDP using a more specific categorical smoking variable (*n*=8,999) – (ii) on Figure 4.

Maternal outcome	Maternal alcohol intake during pregnancy			Adjusted model (binary smoking during pregnancy)		Adjusted model (categorical smoking during pregnancy)	
	Heavy *n* (%)	Low-to-moderate *n* (%)	None *n* (%)	OR[*, †] (95%CI)	*p*-value	OR[*, †] (95%CI)	*p*-value
HDP – *n* complete cases	**1,888**	**4,696**	**2,415**	-	-	-	-
Yes	259 (13.7)	765 (16.3)	466 (19.3)	0.85 (0.78 to 0.92)	<0.001	0.85 (0.78 to 0.92)	<0.001
				RR[†, ‡] (95%CI)	*p*-value	RR[†, ‡] (95%CI)	*p*-value
Gestational hypertension – *n* complete cases	**1,888**	**4,696**	**2,415**	-	-	-	-
Yes	229 (12.1)	681 (14.5)	398 (16.5)	0.86 (0.79 to 0.94)	0.001	0.86 (0.79 to 0.94)	0.001
Preeclampsia – *n* complete cases	**1,888**	**4,696**	**2,415**	-	-	-	-
Yes	30 (1.6)	84 (1.8)	68 (2.8)	0.74 (0.59 to 0.92)	0.007	0.74 (0.59 to 0.92)	0.007

[*] Odds ratio generated using logistic regression

[†] Adjusted for covariates: maternal age at delivery, maternal body mass index (pre-pregnancy), pre-pregnancy smoking (binary), smoking during pregnancy, parity (0, 1, 2 or ≥3), maternal ethnicity (white or non-white), maternal education (32 weeks' gestation) and marital status

[‡] Relative risk ratio generated by multinomial logistic regression

Table S21. Maternal alcohol intake during pregnancy and HDP excluding those who responded after 20 weeks' gestation (n=6,001) – (iii) on Figure 4.

Maternal outcome	Maternal alcohol intake during pregnancy			Unadjusted model		Adjusted model	
	Heavy *n* (%)	Low-to-moderate *n* (%)	None *n* (%)	OR* (95%CI)	*p*-value	OR*, † (95%CI)	*p*-value
HDP – *n* complete cases	1,123	2,887	1,991	-	-	-	-
Yes	156 (13.9)	462 (16.0)	393 (19.7)	0.80 (0.73 to 0.89)	<0.001	0.83 (0.75 to 0.92)	0.001
				RR‡ (95%CI)	*p*-value	RR†, ‡ (95%CI)	*p*-value
Gestational hypertension – *n* complete cases	1,123	2,887	1,991	-	-	-	-
Yes	142 (12.6)	412 (14.3)	343 (17.2)	0.82 (0.74 to 0.91)	<0.001	0.85 (0.76 to 0.95)	0.003
Preeclampsia – *n* complete cases	1,123	2,887	1,991	-	-	-	-
Yes	14 (1.3)	50 (1.7)	50 (2.5)	0.68 (0.51 to 0.89)	0.005	0.72 (0.54 to 0.95)	0.020

* Odds ratio generated using logistic regression

† Adjusted for covariates: maternal age at delivery, maternal body mass index (pre-pregnancy), pre-pregnancy smoking (binary), smoking during pregnancy (binary), parity (0, 1, 2 or ≥3), maternal ethnicity (white or non-white), maternal education (32 weeks' gestation) and marital status

‡ Relative risk ratio generated by multinomial logistic regression

Table S22. Maternal alcohol intake and HDP excluding abstainers prior to pregnancy (*n*=8,450) – (iv) on Figure 4.

Maternal outcome	Maternal alcohol intake during pregnancy			Unadjusted model		Adjusted model	
	Heavy *n* (%)	Low-to-moderate *n* (%)	None *n* (%)	OR* (95%CI)	*p*-value	OR*, † (95%CI)	*p*-value
HDP – *n* complete cases	1,881	4,663	1,906	-	-	-	-
Yes	257 (13.7)	763 (16.4)	375 (19.7)	0.80 (0.74 to 0.88)	<0.001	0.84 (0.77 to 0.92)	<0.001
				RR‡ (95%CI)	*p*-value	RR†, ‡ (95%CI)	*p*-value
Gestational hypertension – *n* complete cases	1,881	4,663	1,906	-	-	-	-
Yes	227 (12.1)	679 (14.6)	321 (16.8)	0.82 (0.75 to 0.90)	<0.001	0.86 (0.78 to 0.94)	0.001
Preeclampsia – *n* complete cases	1,881	4,663	1,906	-	-	-	-
Yes	30 (1.6)	84 (1.8)	54 (2.8)	0.70 (0.56 to 0.89)	0.003	0.76 (0.60 to 0.95)	0.019

* Odds ratio generated using logistic regression
† Adjusted for covariates: maternal age at delivery, maternal body mass index (pre-pregnancy), pre-pregnancy smoking (binary), smoking during pregnancy (binary), parity (0, 1, 2 or ≥3), maternal ethnicity (white or non-white), maternal education (32 weeks' gestation) and marital status
‡ Relative risk ratio generated by multinomial logistic regression
